# A Retrospective Study of the Functional Outcomes in Patients with Proximal Humeral Bone Defect after Shoulder Fusion or Prosthetic Replacement

**DOI:** 10.3390/jcm12113616

**Published:** 2023-05-23

**Authors:** Zhen Pan, Dongdong Cheng, Hua Guo, Zhaohui Li, Xiang Fei, Ting Yuan, Qingcheng Yang

**Affiliations:** Department of Orthopedics, Shanghai Jiao Tong University Affiliated Sixth People’s Hospital, No. 600, Yishan Road, Shanghai 200233, China

**Keywords:** proximal humerus bone tumor, proximal humeral bone defect, arthrodesis, prosthetic replacement, shoulder function

## Abstract

Aims: The reconstruction of proximal humeral defects resulting from tumor resection is challenging. The purpose of this work was to retrospectively study the functional outcomes in patients with large bone defects after the resection of proximal humeral tumors. Methods: We performed a retrospective analysis of 49 patients with malignant or aggressive benign tumors in the proximal humerus at our institution between 2010 and 2021. Forty-nine patients were included in the study (prosthetic replacement, n = 27; shoulder arthrodesis, n = 22). The mean follow-up was 52.8 months (range, 14–129 months). The factors evaluated included the Musculoskeletal Tumor Society (MSTS) functional score, Constant Murley Score (CMS), and complications. Results: Of the 49 patients enrolled in the study, 35 were disease-free by the time of the latest follow-up, and 14 died because of the disease. Adjuvant therapies and medical comorbidities were similar between the two groups. Osteosarcoma was the most common abnormality among all the patients. The mean MSTS scores for surviving patients in the prosthesis and arthrodesis groups were 57.4% and 80.9%, respectively. The mean CMS score for the surviving patients in the prosthesis group was 43.47, and it was 61.44 for arthrodesis cases. Patients with shoulder arthrodesis demonstrated evidence of bony union at a mean of 4.5 months. Conclusions: Shoulder arthrodesis is a reliable reconstructive procedure in patients with large bone defects after the resection of proximal humeral tumors for pediatric osteosarcoma patients. Moreover, prosthetic replacement with anatomical implants results in poor function in older metastasis patients with large bone defects and resection of the deltoid muscle.

## 1. Introduction

The proximal humerus is a common site of malignant tumors, aggressive benign tumors, and metastatic lesions. Some studies have reported that the incidence of humerus malignancies in osteosarcoma was 10%, with 90% of tumors in the proximal humerus [1], and the metastases were mainly located at the proximal end of the femur (59.8%) and in the humerus (18.8%) [2]. The reconstruction of long-bone defects after tumor resection poses a great challenge for surgeons. Limb salvage and reconstructive surgeries have become common treatment approaches for tumors of the proximal humerus due to advances in chemotherapy, improvements in imaging techniques, and newer surgical procedures. Shoulder reconstruction after tumor resection may involve allografts, autografts, prosthetic devices, and arthrodesis [3].

Prosthetic reconstruction is an established and common treatment for shoulder girdle tumors, with a relatively simple procedure and immediate stability [4]. However, shoulder joint motion with prosthetic reconstruction after resection of the rotator cuff and deltoid is always very limited postoperatively, and the prosthesis is likely to loosen and deteriorate with time, especially in young patients who are expected to have long-term survival and engage in strenuous activity [5].

High-grade sarcomas require wide resection of the shoulder, including the rotator cuff and deltoid muscles, to ensure adequate surgical margins. The lack of these functional muscles results in severely restricted shoulder movements. Therefore, prosthetic replacement does not provide satisfactory postoperative shoulder joint function. With the improvements in surgical methods and techniques, biological reconstruction has been gradually performed and improved long-term functionality. Scapulohumeral arthrodesis using an autogenous free vascularized fibular graft (FVFG) has been reported to be an appropriate salvage procedure with satisfactory functional outcomes [6]. The features of arthrodesis are the restoration of shoulder skeletal stability and long-term durable reconstruction, even after excision of the surrounding functional muscles [7].

Fibular grafting is a popular method for the reconstruction of segmental defects after tumor resection [8,9]. Arthrodesis using FVFG is demanding and challenging. Fuchs et al. [3] reported a large number of patients who had acceptable functional outcomes after tumor resection followed by shoulder arthrodesis using FVFG. Gebert et al. [10] reviewed 21 patients who underwent FVFG for diaphyseal defect reconstruction of the upper extremity and concluded that the procedure has broader structural applications.

In the present work, we retrospectively study the functional outcomes in patients with large bone defects after the resection of proximal humeral tumors. We aimed to determine the clinical outcome of shoulder arthrodesis and prosthetic replacement in the reconstruction of bone defects and identify whether the FVFG technique could be effectively applied for the reconstruction of bone defects.

## 2. Patients and Methods

### 2.1. Clinical Series

Between 2010 and 2021, 49 patients with malignant, aggressive, benign, or metastatic tumors of the proximal humerus underwent limb salvage surgeries at our cancer center. Nineteen patients were men and 30 were women. The average age at the time of surgery was 40.2 years (range 8–80 years). This study was approved and monitored by the Human Research Ethics Committee of our hospital. All patients provided written informed consent to participate in the study, as well as to include their details and images in publications.

This study included only patients with tumors in the proximal aspect of the humerus, and preoperative imaging that showed a satisfactory margin could be achieved. Patients with a tumor involving the clavicle, scapula, neurovascular bundle, or the proximal part of the humeral diaphysis (without involvement of the humeral head) were excluded. There were 20 osteosarcomas, 9 chondrosarcomas, 12 metastases, and 8 other sarcomas (Table 1). Pathological fractures were found in 2 patients preoperatively. Open or core needle biopsies were performed in all patients before surgery.

Twenty patients with osteosarcoma and 12 with metastatic disease received chemotherapy before surgical reconstruction; one patient with hemangiosarcoma did not receive preoperative chemotherapy because he had a pathological fracture at the initial presentation. Postoperative chemotherapy was administered to 32 patients. The other patients with chondrosarcoma and giant cell tumors did not receive chemotherapy.

### 2.2. Resection and Reconstructive Procedures

Resection margins were determined based on preoperative magnetic MRI, CT, bone scan, and plain radiography. Intraoperative frozen-section analysis was performed to ensure a negative surgical margin.

### 2.3. Tumor Resection

All surgeries were performed using an extended deltopectoral approach that included a previous biopsy track. The deltoid, long head of the biceps, latissimus teres major complex, and rotator cuff were released from their insertions in the humerus. A humeral tumor with a normal cuff of the surrounding muscle was isolated and cut at least 2 cm from the most distal part of the lesion (Supplemental Figure S1). Specimens of the distal bone marrow and clinically worrisome soft tissue margins were evaluated using frozen-section microscopic examination.

### 2.4. Prosthetic Replacement

Prosthesis reconstruction was performed on 27 patients. After tumor removal, the humeral canal was reamed, and a modular or custom proximal humeral prosthesis was inserted with cement. The prosthetic humeral head must face slightly backward (35–40° retroversion) to articulate with the glenoid. The surrounding soft tissue was reattached to the appropriate insertion of the prosthesis (Supplemental Figure S2A). 

### 2.5. Shoulder Arthrodesis

**Figure 1:**
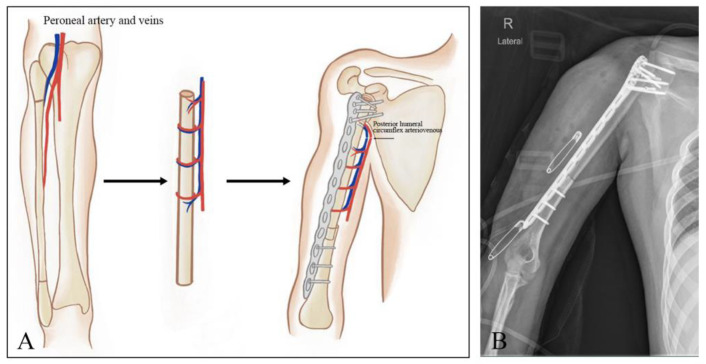
Schematic diagram illustrating the surgical procedures. (**A**) Preparation of the free vascularized fibula graft and reconstruction of the humeral defects with the fibular graft placed in the medullary canal of a cortical allograft, followed by vascular anastomosis. (**B**) Shoulder arthrodesis radiographic appearance of the proximal humeral prosthesis replacement.

Twenty-two patients underwent shoulder arthrodesis. Further, there were 8 patients for whom FVFG was used. During the tumor resection, a different microsurgical team harvested the free fibular graft. Briefly, the fibula was dissected together with the peroneal artery and veins using a lateral approach. The proximal and distal osteotomy sites were retained for the neck of the fibula and ankle joint stability, respectively. Generally, the fibular graft was longer than the resection length of the humeral defect by 4–5 cm to ensure that the graft could be inserted into the distal end of the humeral bone defect. After reaming the distal humeral canal and removing the articular cartilage from the glenoid fossa in the proximal end, the fibula was inserted approximately 2 cm into the humerus, and the proximal end was fixed with the glenoid using a long, broad 4.5 mm locking compression plate. Screws were placed as deeply as possible into the scapular body to enhance stability, and at least one screw was placed into the scapular spine. The preferred position of the fused shoulder was 20–30° of abduction, 30° of forward flexion, and 45° of internal rotation. A vascular anastomosis was then performed. Using routine microsurgical techniques, the graft vascular pedicle was anastomosed to the posterior humeral circumflex artery. A diagram illustrating the surgical procedures is depicted in Figure 1.

### 2.6. Postoperative Care

All patients with prosthetic replacement or shoulder arthrodesis were kept in a sling for at least 4 weeks to allow healing of the soft tissue. Active elbow and hand motion was initiated in the immediate postoperative period. After 4 weeks, slow progressive shoulder exercises were initiated in the patients treated with prostheses. Patients with arthrodesis were kept in a sling until osseous union was confirmed on radiography. 

### 2.7. Functional Assessment

Postoperative function was evaluated according to the Musculoskeletal Tumor Society (MSTS) rating system [11], which includes pain, function, emotional acceptance, hand positioning, manual dexterity, and lifting ability. Each category was scored from 0 to 5, with a maximum total score of 30. The total score was then converted to a percentage (raw score/30 × 100). Shoulder motion range measurements were recorded using the Constant Murley Score (CMS), which includes pain, activities of daily living, range of motion, and strength. The MSTS and CMS scores for each patient were completed by a surgeon as part of the follow-up examination.

## 3. Results

### 3.1. Summary of Demographic Data 

**Figure 2:**
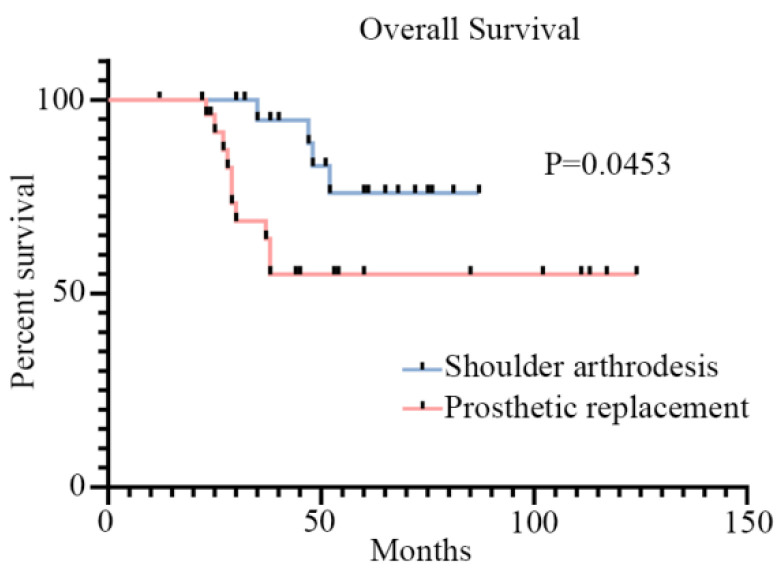
The overall survival of patients between the shoulder arthrodesis group and the prosthetic replacement group. Data are shown as the means ± SD, and statistical analysis was performed using the log-rank test. Error bars represent the SEM.

The baseline characteristics of the patients are summarized in Table 1. Forty-nine patients were included in the study (prosthetic replacement, n = 27; shoulder arthrodesis using FVFG, n = 22). There were 3 men and 19 women, with an average age of 28.3 years, in the shoulder arthrodesis group using FVFG. The prosthetic replacement group included 16 men and 11 women, with an average age of 49.9 years. As regards the histological diagnosis, there were 14 osteosarcomas, 3 chondrosarcomas, 2 metastases, and 3 other types in the shoulder arthrodesis group using FVFG. There were 6 osteosarcomas, 6 chondrosarcomas, 10 metastases, and 5 other types in the prosthetic replacement group. In the FVFG group, 16 patients had a tumor necrosis rate <90%, and in 6, the rate was ≥90. Likewise, there were 20 patients with a tumor necrosis rate <90% and 7 with ≥90 in the prosthetic replacement group. There were 6 cases of low-grade tumors (AJCC/TNM stage I, IIA) and 16 cases of high-grade tumors (AJCC/TNM stage IIB, III, and IV) in the FVFG group. In the prosthetic replacement group, there were 8 cases of low-grade tumors (AJCC/TNM stage I, IIA) and 19 cases of high-grade tumors (AJCC/TNM stage IIB, III, and IV). The 5-year survival rate in the FVFG group was 81.8%, which was higher than that in the prosthetic replacement group (63.0%). In addition, the Kaplan–Meier survival curve showed that patients with shoulder arthrodesis using FVFG had a longer overall survival time than those in the prosthetic replacement group (Figure 2). 

### 3.2. Oncological Evaluation

The classification of surgical stages of tumors was based on the Enneking system [12]. All patients underwent wide resection (type IB) according to the scheme proposed by Malawer [13]. 

The mean follow-up was 52.8 months. Three patients with osteosarcoma died of pulmonary metastasis and one died of lung cancer in the shoulder arthrodesis group. In addition, four patients with chondrosarcoma and osteosarcoma died of pulmonary metastasis, and six died of primary cancer in the prosthetic replacement group. In the shoulder arthrodesis group, the mean length of the humeral defect was 11 cm (range, 8–18 cm) and the fibula bone measured 15 cm (range, 12–22 cm). The average numbers of proximal and distal screws for internal fixation in this group were five and four, respectively. The mean humeral defect length in the prosthetic replacement group was 16 cm (range, 8–28 cm). Postoperative histological examination revealed clear surgical margins in all the patients (Table 2 and Table 3).

### 3.3. Arthrodesis Union and Healing

**Figure 3:**
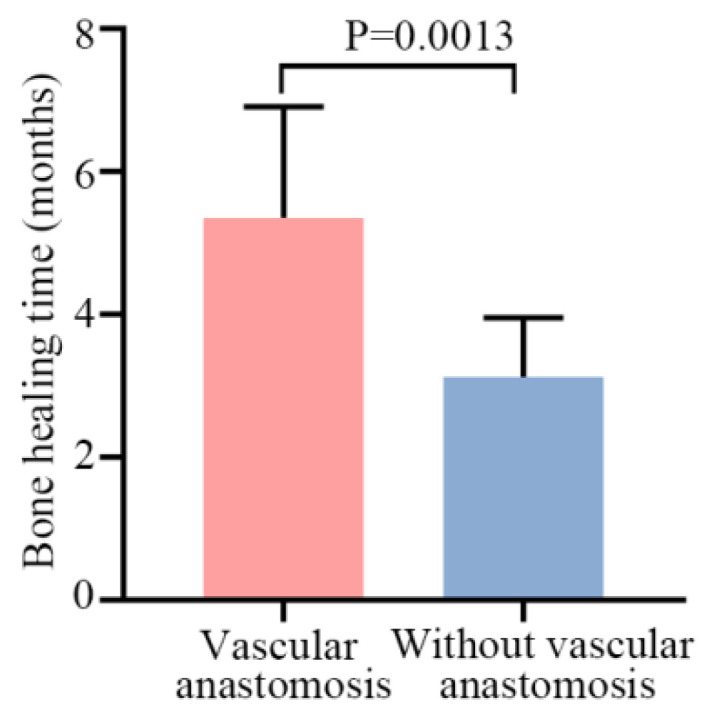
The bone healing times in the patients with vascular anastomosis and without vascular anastomosis. Data are shown as the means ± SD, and statistical analysis was performed using Student’s *t*-test. Error bars represent the SEM.

Bone union in all patients with shoulder arthrodesis was confirmed using radiography at a mean of 4.5 months (range, 3–8 months; Table 2). Eight patients (age < 17 years) in the shoulder arthrodesis group used a vascularized fibular graft. The results showed that bone healing times in patients with vascular anastomosis were shorter than those in patients without it (Figure 3). In two patients, the screw at the proximal fibular graft–glenoid interface was fractured in 1–2 years, and arthrodesis was healed. However, the patients did not require any further surgical interventions. In one patient, due to pulmonary metastasis and local recurrence of the tumor, amputation had to be performed.

### 3.4. Clinical Outcome

**Figure 4:**
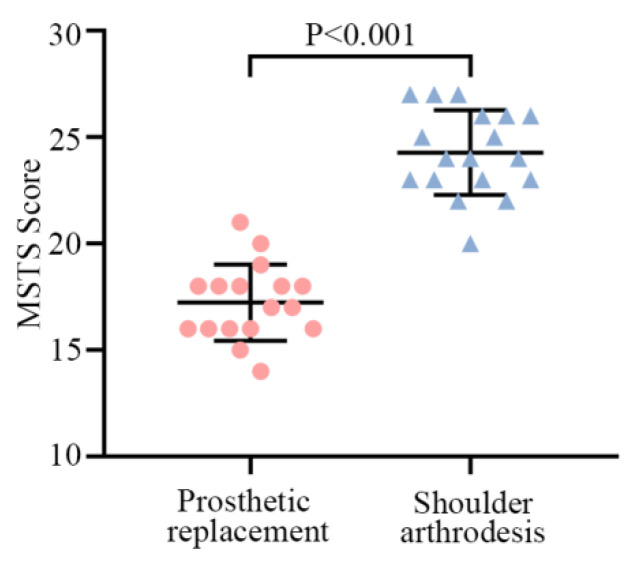
The MSTS scores of patients between the shoulder arthrodesis group and the prosthetic replacement group. Data are shown as the means ± SD, and statistical analysis was performed using Student’s *t*-test. Error bars represent the SEM.

Excluding the 10 patients who died within 3 years postoperatively in the prosthesis group, the mean MSTS score for the remaining 17 patients was 57.4% (range, 46.7–70%; Table 3 and Table S1). The mean functional MSTS score for cases in the shoulder arthrodesis was 80.9% (range, 66.7–90%), excluding the four patients who died within 3 years postoperatively. With the exception of hand positioning, other categories were rated as satisfactory (>3 points) by patients in the shoulder arthrodesis group (Table 2 and Table S1). The MSTS score was higher in the shoulder arthrodesis group than that in the prosthesis group (Figure 4). Furthermore, patients with fused shoulders had much better function and lifting ability (Supplemental Figures S2 and S3).

**Figure 5:**
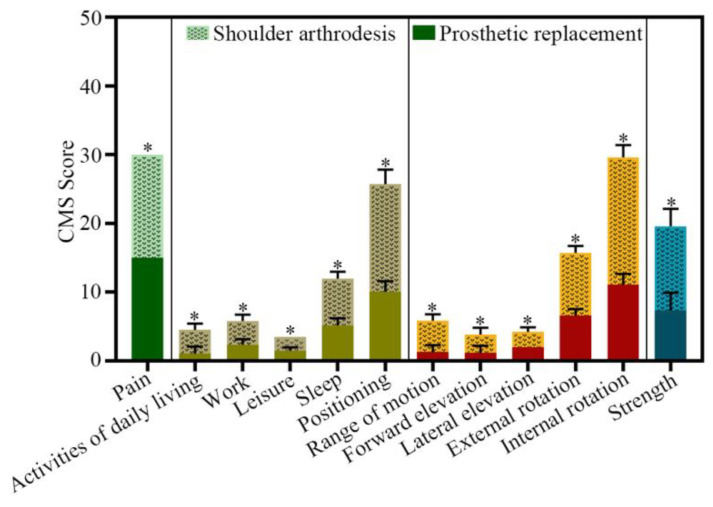
The range of CMS scores between the shoulder arthrodesis group and the prosthetic replacement group. Data are shown as the means ± SD, and statistical analysis was performed using Student’s *t*-test. Error bars represent the SEM. * *p* < 0.05.

The CMS questionnaires, including the constructs and their subscales, are shown in Figure 5. It is clear that the mean scores in the shoulder arthrodesis group were higher than those in the prosthesis group. In the prosthesis group, the results showed that patients could reach their chest comfortably and judged their activities of daily living to be moderately difficult (1–2 of four points). All patients achieved <60° lateral elevation (range, 30–60°) and forward flexion (range, 30–60°) and the adductor muscle strength scores were <grade II on average (Table 4 and Table S2). Meanwhile, in the shoulder arthrodesis group, patients were able to reach the head and neck comfortably and judged their activities of daily living to be slightly impaired or not at all (2–4 of four points). Unlike the patients in the prosthesis group, all patients achieved >60° of forward flexion (range, 60–120°) and lateral elevation (range, 60–90°); the adductor muscle strength score reached grade III (Table 4 and Table S2). Additionally, none of the patients experienced painful scapular winging due to excessive abduction or flexion with the arm in a resting position. Collectively, these results showed that shoulder function in the shoulder arthrodesis group was better than that in the prosthesis group.

## 4. Complications

Of the 27 patients who underwent reconstruction with a proximal humeral prosthesis, two had subluxation of the implant (Cases 19 and 23); no local recurrences or deep infections were observed in the remaining patients. Loosening of a lag screw was seen at the proximal fibular graft–glenoid interface in one patient (Case 18) 6 months after surgery, probably due to excessive movement of the shoulder. Radiographic osseous union was observed at 2 months, and the patient was treated conservatively under observation. No complications occurred at the fibular donor site. None of the patients in this study required additional bone grafting to achieve arthrodesis. Furthermore, no intraoperative complications, donor site morbidity, or venous thrombosis were observed.

## 5. Discussion

With the development of neoadjuvant chemotherapy, the survival rates of patients with malignant tumors, aggressive benign tumors, and metastatic lesions have greatly improved in the past few decades. Therefore, new challenges have been presented to oncological surgeons. The reconstructed shoulder after tumor resection is required to be not only cosmetic and functional, but also useful in the long-term, as well as durable for strenuous activity. Reconstruction after proximal humerus resection for tumors is still challenging, despite the different techniques proposed.

Several reconstructive procedures have been reported to provide satisfactory shoulder function after wide resection of the tumor [14,15]. Prosthetic reconstruction is simpler than other reconstructions. The duration of prosthetic reconstruction was approximately 2 h in this study, which was less than half that of the other two biological procedures. Although reconstruction with a proximal humeral prosthesis could stabilize the upper extremity to the glenoid cavity and shoulder acromion to enable shoulder motion, slight shoulder function was restored postoperatively. The patients with prostheses in the present study had satisfactory pain release, and their daily routine was moderately difficult. Meanwhile, the MSTS and CMS scores for hand positioning were compromised, and the lifting ability and function were very low. Furthermore, the motion of the treated shoulder was grossly limited, even with preservation of the axillary nerve and rotator cuff, by attachment to the prosthesis. The lateral elevation and forward flexion ranges in the prosthetic replacement group were lower than those in the shoulder arthrodesis group. No more than 30° abduction and 45° forward flexion could be achieved in prosthesis cases in the present study and in most published studies [15,16,17].

Presently, shoulder arthrodesis is indicated for brachial plexus injury, failed prosthetic arthroplasty, reconstruction after tumor resection, chronic infection, refractory instability, and pseudoparalysis of the shoulder secondary to combined rotator cuff and deltoid muscle dysfunction [18]. Shoulder arthrodesis is a biological arthroplasty that transfers the site of motion from the glenohumeral joint to the scapulothoracic “joint” [6]. In the presence of severe proximal humeral deficiency, bone stock could be substituted for various techniques, such as the use of structural tricortical bone grafts or allografts [18]. However, it is difficult to consider biological reconstruction in the presence of extensive humeral bone loss. When the proximal humerus presents a deficiency of ≥ 6 cm, shoulder arthrodesis with FVFG might have an acceptable outcome as a salvage procedure [4,6]. In the last century, Taylor et al. [19] first reported a free vascularized fibular autograft for the restoration of large long bone defects. It has also been reported that a graft of up to 26 cm in length could be acquired from the fibula [20]. Therefore, FVFG could be considered a better option for the restoration of bone defects in both the upper and lower extremities. Shoulder arthrodesis should provide a stable shoulder girdle for routine activities, albeit with limited motion. With the development of current microsurgical techniques, it is more feasible to transfer the fibula to recipient sites, which in turn increases graft survival. Xu et al. [21] reported the results of a series of osteosarcoma patients who underwent biological reconstruction using FVFG after tumor resection. The authors suggested that FVFG could yield acceptable overall outcomes accompanied by a high union rate and patient satisfaction.

In this study, patients with arthrodesis had better functional MSTS scores (80.9%). A wider range of abduction and flexion (>60°) of the treated shoulder was obtained in all patients, in marked contrast with those with prostheses. In addition, patients in the arthrodesis group reported no pain, accomplished strenuous activities of daily living, and reached the head and neck comfortably. The mean time to union was 4.5 months. Meanwhile, the bone union time for teenagers with vascular anastomosis was shorter than that of adults without vascular anastomosis. Xu et al. [21] reported 18 patients who underwent tumor resection with the use of FVFG. The average time to union was 4.5 months, which is comparable to that of the patients in the present study.

By comparing these data, we also found some problems. Most arthrodesis procedures were performed on pediatric osteosarcoma patients, whereas most prosthetic replacements were performed on older patients with metastases. The reasons could be that surgery for prosthetic replacement is more common in the early stage. With the progression of time and improvements in surgical methods and techniques, shoulder arthrodesis has been gradually performed. Moreover, older patients with metastases, whose survival is shorter, prefer simple prosthetic replacement. In contrast, for pediatric patients with osteosarcoma of the proximal humerus, their prognosis is good and their postoperative survival is longer. Although the surgery to reconstruct the limb function is complicated, performing the limb function reconstruction surgery on them is more beneficial for the recovery of shoulder function.

In addition, shoulder abduction function was better with shoulder arthrodesis in this study. Restoring anatomy and shoulder function after proximal humerus resection for a bone tumor is demanding. In the early stages, for patients with tumors with large proximal humeral bone defects (especially for older patients with metastases), we could only choose prosthetic replacement. For these patients, the deltoid muscle had been removed and shoulder function was dependent on the rotator cuff and its surrounding soft tissues. This resulted in poorer function (especially abduction function). For pediatric patients with osteosarcoma of the proximal humerus, they were treated with shoulder arthrodesis, as their prognosis was good and their postoperative survival was longer. In addition, the shoulder joint was driven by the scapula after the shoulder arthrodesis. The range of motion of the shoulder joint was greater with bone structure compared to the soft tissue [6].

According to Charles Sumner Neer II, shoulder hemiarthroplasty was intended to ease local pain, preserve the normal anatomy of the site, and meanwhile provide sufficient functionality to the involved upper limb [22]. Osteoarticular allografts, bone autografts, prostheses, and graft prothesis composites represent the current treatments of choice for proximal humerus reconstruction. All the aforementioned solutions guarantee the static repair of the shoulder in association with at least partial restoration of its articularity. The most suitable approach can vary depending on the patients’ age, clinical picture (considering comorbidities as well as life expectancy), functional demands, and the eventual sacrifice of the rotator cuff, deltoid muscle, or axillary nerve through the procedure [23,24,25]. With the advancement of technology, the design of prostheses is becoming more and more compatible with the anatomical structure of the shoulder. However, due to their mediocre functional outcomes, anatomical endoprostheses are not to be considered among the most suitable approaches for young, high-demand patients. On the contrary, these reconstructions represent reliable options for patients of older age, with lower functional requests, and/or suffering from systemic diseases [26]. In addition, there are studies reporting that modular reverse total shoulder arthroplasty appears to be a reasonable reconstruction option after proximal humerus resections if an innervated deltoid muscle is spared, especially for those patients who expect to survive for longer than 1 year [27]. This reconstructive procedure combines the advantages of biological reconstruction with those of prosthetic reconstruction.

In the current study, the most severe complication was subluxation of the implant in two cases and screw loosening in one case. Patients with shoulder arthrodesis tended to have better function and improved abilities to perform daily activities. Patients in the shoulder arthrodesis group did not require more soft tissue coverage and had significantly fewer complications. Nevertheless, the study had a few limitations. First, this was a small retrospective case series, and statistical analysis between the groups was insufficient. The patients who underwent vascular anastomosis were children, whose bones heal better than those of adults. Second, because the choice of reconstruction for patients with tumors remained controversial, the techniques were chosen based on our experience, and not on randomization. All the reconstruction procedures were performed using the same protocol. Therefore, the variability in surgical techniques was minimized.

## 6. Conclusions

The clinical outcomes of this study indicated that shoulder arthrodesis using FVFG was effective for local tumor control and the preservation of shoulder joint movement. Shoulder arthrodesis provided a stable shoulder girdle with satisfactory function and acceptable complication rates. The bone healing times in patients with vascular anastomosis were shorter. Thus, it could be stated that the FVFG technique could be effectively applied for the reconstruction of bone defects.

## Figures and Tables

**Table 1:** Demographic data of the patients for two commonly used reconstructive techniques, prosthetic replacement and glenohumeral joint arthrodesis using free vascularized fibular graft (FVFG).

Types	Shoulder Arthrodesis Using FVFG	Prosthetic Replacement
Gender		
Male	3	16
Female	19	11
Age (years)		
<20	9	2
≥20	13	25
Average	28.3	49.9
Histological diagnosis		
Osteosarcoma	14	6
Chondrosarcomas	3	6
Metastases	2	10
Others	3	5
Tumor necrosis rate (%)		
<90	16	20
≥90	6	7
AJCC/TNM stage		
I/IIA	6	8
IIB/III/IV	16	19
5-year survival rate (%)	81.8%	63.0%

**Table 2:** Details of patients who underwent glenohumeral joint arthrodesis using free vascularized fibular graft (FVFG). M, male; F, female; MSTS, musculoskeletal tumor society; Y, vascular anastomosis; N, without vascular anastomosis; DF, disease-free.

Case	Gender/Age	Histological Diagnosis	MSTS Score %	Vascular Anastomosis	Bone Healing Time (Months)	Length of Humeral Defect (cm)	Length of Fibula Bone (cm)	Number of Proximal/Distal Screws for Internal Fixation	Outcomes	Follow-Up (Months)
1	F/19	osteosarcoma	90.0	N	5	12	16	4/4	DF	65
2	F/14	osteosarcoma	90.0	Y	3	10	14	7/5	DF	60
3	F/28	osteosarcoma	83.3	N	4	14	18	5/5	DF	40
4	F/38	hemangiosarcoma	86.7	N	6	8	12	6/5	DF	38
5	F/11	osteosarcoma	86.7	Y	3	17	21	5/3	DF	32
6	M/14	osteosarcoma	/	Y	3	15	19	7/3	Death	35
7	F/22	osteosarcoma	90.0	N	4	8	12	6/4	DF	51
8	F/23	intermediate fibrohistiocytoma	86.7	N	4	8	12	5/3	DF	72
9	F/50	chondrosarcoma	73.3	N	8	8	12	5/4	DF	48
10	M/14	osteosarcoma	80.0	Y	5	13	17	5/5	DF	30
11	F/33	giant cell tumor	76.7	N	5	8	12	7/4	DF	68
12	F/17	osteosarcoma	80.0	Y	3	12	16	4/3	DF	87
13	F/49	metastases (lung cancer)	76.7	N	7	10	14	3/3	DF	75
14	F/63	osteosarcoma	/	N	8	8	12	6/3	Death	52
15	F/9	osteosarcoma	/	Y	3	12	16	4/4	Death	48
16	F/32	osteosarcoma	83.3	N	4	9	13	6/3	DF	81
17	F/42	chondrosarcoma	73.3	N	5	8	12	5/3	DF	76
18	F/14	osteosarcoma	76.7	Y	2	10	14	5/5	DF	72
19	F/73	metastases (lung cancer)	/	N	7	18	22	6/3	Death	47
20	F/28	chondrosarcoma	76.7	N	4	8	12	8/6	DF	61
21	M/20	osteosarcoma	66.7	N	4	16	20	5/6	DF	12
22	F/10	osteosarcoma	80.0	Y	3	11	15	5/3	DF	75
Average	28.30	/	80.9	/	4.5	11	15	5/4	-	55.68

**Table 3:** Details of patients who underwent prosthetic replacement.

Case	Gender/Age	Histological Diagnosis	MSTS Score %	Length of Humeral Defect (cm)	Outcomes	Follow-Up (Months)
1	M/42	chondrosarcoma	-	14	Death	29
2	M/22	osteosarcoma	-	16	Death	23
3	F/49	metastases (kidney cancer)	-	14	Death	38
4	M/55	malignant fibrohistiocytoma	60.0	16	DF	124
5	M/32	giant cell tumor	60.0	14	DF	117
6	M/60	osteosarcoma	-	12	Death	38
7	F/18	osteosarcoma	70.0	14	DF	113
8	M/40	chondrosarcoma	60.0	16	DF	111
9	M/40	metastases (liver cancer)	-	14	Death	29
10	F/25	osteofibrous dysplasia	66.7	18	DF	102
11	M/58	chondrosarcoma	-	14	Death	27
12	M/60	metastases (liver cancer)	-	26	Death	37
13	M/58	chondroma	63.3	8	DF	85
14	M/66	chondrosarcoma	60.0	16	DF	60
15	F/62	metastases (lung cancer)	-	28	Death	25
16	M/54	osteosarcoma	56.7	16	DF	53
17	F/67	chondrosarcoma	60.0	14	DF	54
18	F/68	metastases (lung cancer)	-	14	Death	28
19	F/50	undifferentiated pleomorphic sarcoma	53.3	26	DF	45
20	M/59	metastases (kidney cancer)	-	14	Death	30
21	F/65	chondrosarcoma	46.7	24	DF	44
22	F/65	metastases (thyroid cancer)	53.3	14	DF	38
23	M/21	osteosarcoma	56.7	18	DF	24
24	F/8	osteosarcoma	53.3	22	DF	23
25	M/62	metastases (kidney cancer)	53.3	12	DF	23
26	F/63	metastases (lung cancer)	50.0	10	DF	23
27	M/80	metastases (lung cancer)	53.3	14	DF	22
Average	49.90	-	57.4	16	-	50.55

M, male; F, female; MSTS, musculoskeletal tumor society; DF, disease-free.

**Table 4:** Principal component analysis of CMS.

CMS Items	Prosthetic Replacement				Free Vascularized Fibular Graft (FVFG)			
	Min	Max	Mean	SD	Min	Max	Mean	SD
Pain	15	15	15	0	15	15	15	0
Activities of daily living	7	12	10.06	1.52	12	18	15.67	2.09
Work	0	2	1.06	1.03	2	4	3.44	0.92
Leisure	2	4	2.35	0.76	2	4	3.44	0.92
Sleep	1	2	1.47	0.51	2	2	2	0
Positioning	4	6	5.18	1.01	6	8	6.78	1.00
Range of motion	8	12	11.06	0.94	16	24	18.56	1.79
Forward elevation	0	2	1.29	0.99	4	6	4.56	0.92
Lateral elevation	0	2	1.18	1.01	2	4	2.67	0.97
External rotation	2	2	2	0	2	4	2.22	0.65
Internal rotation	4	8	6.59	0.94	8	10	9.11	1.02
Strength	5	10	7.35	2.57	10	15	12.22	2.56
Sum of the score	-	-	61.44	4.58	-	-	43.47	2.58

## Data Availability

Due to the nature of this research, participants of this study did not agree for their data to be shared publicly, so supporting data is not available.

## Supplementary Materials

The following supporting information can be downloaded at: https://www.mdpi.com/article/10.3390/jcm12113616/s1.
