# Supplementary material for: A Retrospective Study of the Functional Outcomes in Patients with Proximal Humeral Bone Defect after Shoulder Fusion or Prosthetic Replacement
Source: J Clin Med. 2023 May 23;12(11):3616. doi: 10.3390/jcm12113616 (PMC10254032; doi:10.3390/jcm12113616)
Supplement: Supplementary file 1 [file jcm-12-03616-s001.zip › jcm-2119592-supplementary.pdf]

Supplementary Figure 1

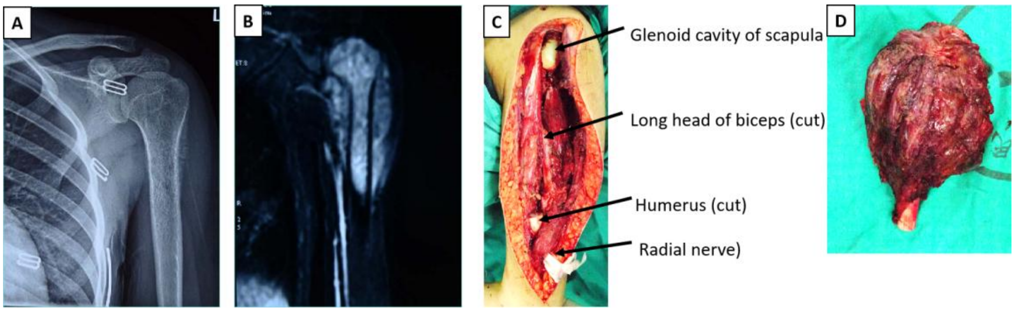

Supplementary Figure 1 X-ray (A) and T2 MRI (B) showing proximal humeral tumor. (C) Surgical resection of type IB was performed. (D) Gross specimen showing the proximal humeral sarcoma covered by its adjacent musculature.

Supplementary Figure 2

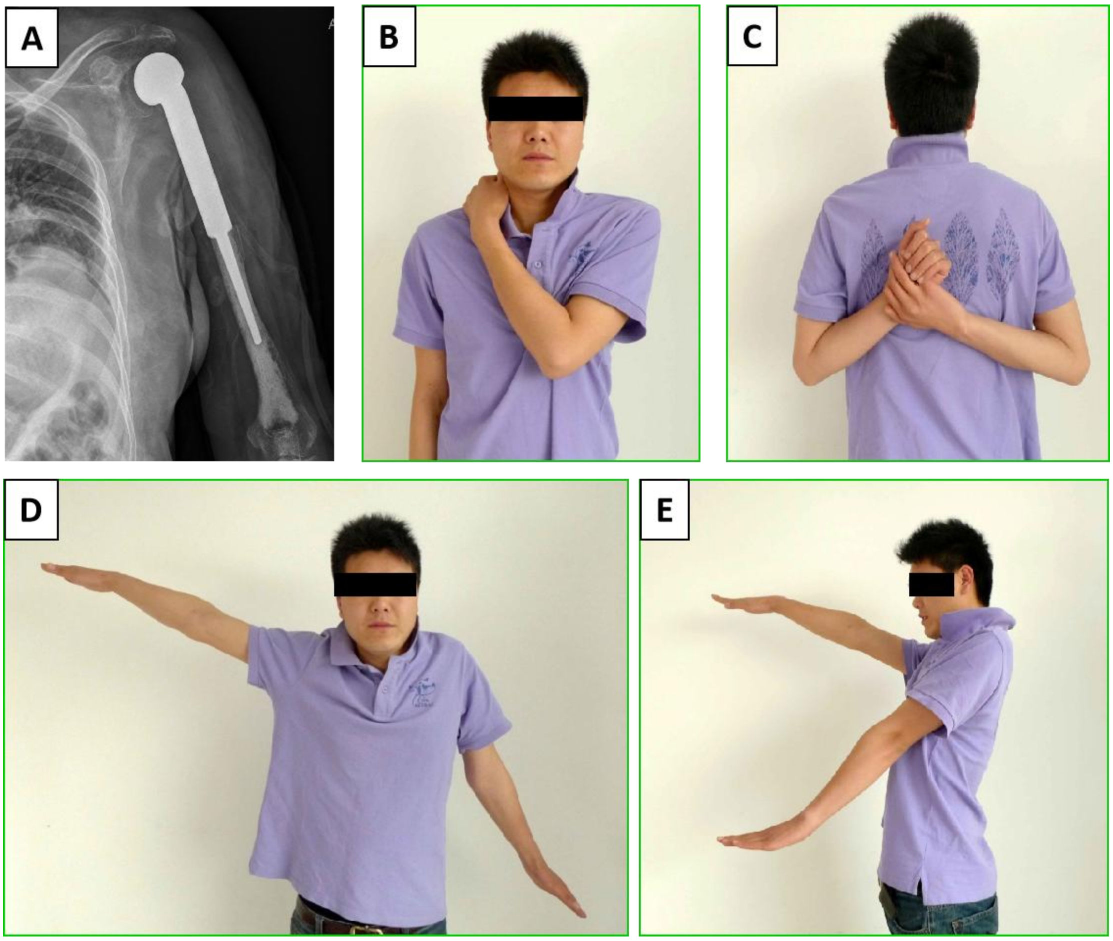

Supplementary Figure 2 A patient undergoing prosthetic replacement of humerus defect with 2.5 year follow-up. (A) A male patient, aged 40 years, was histologically diagnosed as chondrosarcomas located in the humerus. Postoperative radiographic appearance of the proximal humeral prosthesis replacement. (B-E) Function after 2-year follow-up. The patients with prosthesis replacement could reach the other shoulder, the midline of the back. Abduction was 30 degrees, and forward flexion was 45 degrees.

Supplementary Figure 3

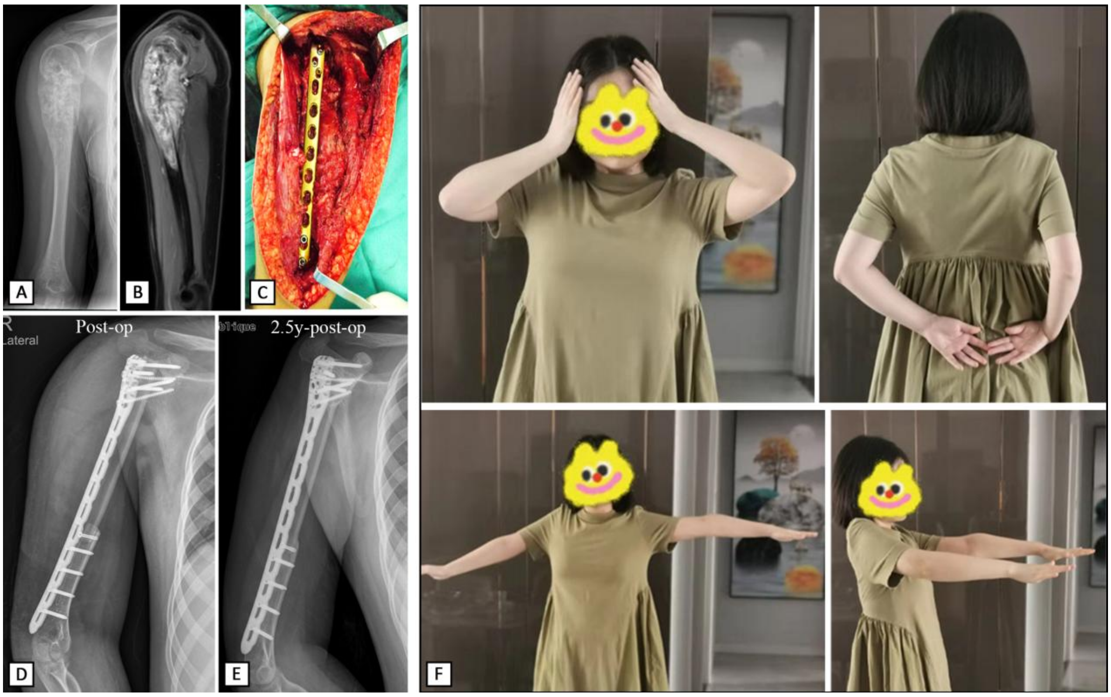

Supplementary Figure 3 A patient undergoing biological reconstruction of humerus defect with 2.5 year follow-up. (A, B) A Female patient, aged 14 years, was histologically diagnosed as osteosarcoma located in the humerus. (C-D) Free vascularized fifibula graft was directly applied to the reconstruction of the bone defect. (E) At the 2.5 year-follow-up, the radiograph indicated complete bone union. No complication was reported. (F) The patient had an excellent functional outcome with a MSTS score of 90%. The patient had an excellent shoulder function and could manage even demanding activities of daily living very comfortably. The patient could reach her hair, the midline of the back, with 85 degrees of shoulder abduction and 85 degrees of forward flexion.

Supplementary Table 1. MSTS functional scores of the cases in two groups.

Case	Pain	Emotional acceptance	Function	Hand positioning	Manual dexterity	Lifting ability	Overall	Percentage* (%)
Shoulder arthrodesis using FVFG								
1	5	5	5	3	5	4	27	90.0
2	5	5	5	3	5	4	27	90.0
3	5	5	5	2	4	4	25	83.3
4	5	4	5	3	5	4	26	86.7
5	5	5	5	3	4	4	26	86.7
6	-	-	-	-	-	-	-	-
7	5	4	5	4	5	4	27	90.0
8	5	4	5	3	5	4	26	86.7
9	5	4	5	2	3	3	22	73.3
10	5	4	5	3	4	3	24	80.0
11	5	3	5	3	4	3	23	76.7
12	5	4	5	3	4	3	24	80.0

13	4	4	5	3	4	3	23	76.7
14	-	-	-	-	-	-	-	-
15	-	-	-	-	-	-	-	-
16	5	4	5	3	4	4	25	83.3
17	4	4	5	2	4	3	22	73.3
18	4	4	5	3	4	3	23	76.7
19	-	-	-	-	-	-	-	-
20	5	4	4	3	4	3	23	76.7
21	4	3	5	1	4	3	20	66.7
22	5	4	5	2	4	4	24	80.0
Prosthetic replacement								
1	-	-	-	-	-	-	-	-
2	-	-	-	-	-	-	-	-
3	-	-	-	-	-	-	-	-
4	5	2	3	3	3	2	18	60
5	5	3	2	3	2	3	18	60
6								
7	5	3	3	3	4	3	21	70
8	5	3	3	2	3	2	18	60
9	-	-	-	-	-	-	-	-
10	5	3	3	3	3	3	20	66.7
11	-	-	-	-	-	-	-	-
12	-	-	-	-	-	-	-	-
13	5	2	3	3	3	3	19	63.3
14	5	2	3	3	3	2	18	60
15	-	-	-	-	-	-	-	-
16	5	2	3	2	3	2	17	56.7
17	5	2	3	3	3	2	18	60
18	-	-	-	-	-	-	-	-
19	5	2	2	2	3	2	16	53.3
20	-	-	-	-	-	-	-	-
21	5	1	2	1	3	2	14	46.7
22	5	1	3	2	3	2	16	53.3
23	5	2	3	2	3	2	17	56.7
24	5	2	2	2	3	2	16	53.3
25	5	2	2	2	3	2	16	53.3
26	5	1	2	2	3	2	15	50
27	5	1	3	2	3	2	16	53.3

*Percentage(%): the ratio of overall MSTS score to total score (30), and each item is 5 points.

Supplementary Table 2. CMS functional scores of the cases in two groups.

Case	Pain	Activities				Range of motion				Strength
		Work	Leisure	Sleep	Positioning	Forward elevation	Lateral elevation	External rotation	Internal rotation	
score	15	4	4	2	10	10	10	10	10	25
Shoulder arthrodesis using FVFG										
1	15	4	4	2	8	4	4	2	10	10
2	15	4	4	2	8	4	4	4	10	15
3	15	4	4	2	6	4	2	2	10	10
4	15	4	4	2	6	4	2	2	10	15
5	15	4	4	2	8	6	2	2	10	10
6	-	-	-	-	-	-	-	-	-	-
7	15	4	4	2	8	6	2	2	10	10
8	15	4	4	2	6	4	4	4	8	10
9	15	2	2	2	6	4	4	2	8	15
10	15	2	4	2	6	4	4	2	8	10
11	15	4	2	2	8	4	2	2	8	10
12	15	4	4	2	6	4	2	2	10	15
13	15	2	2	2	6	4	2	2	8	10
14	-	-	-	-	-	-	-	-	-	-
15	-	-	-	-	-	-	-	-	-	-
16	15	2	2	2	6	4	2	2	8	15
17	15	2	2	2	8	4	2	2	8	10
18	15	4	4	2	6	4	4	2	8	10
19	-	-	-	-	-	-	-	-	-	-
20	15	4	4	2	8	6	2	2	10	15
21	15	4	4	2	6	6	2	2	10	15
22	15	4	4	2	6	6	2	2	10	15
Prosthetic replacement										
1	-	-	-	-	-	-	-	-	-	-
2	-	-	-	-	-	-	-	-	-	-
3	-	-	-	-	-	-	-	-	-	-
4	15	0	2	1	4	2	2	2	8	10
5	15	2	2	2	6	0	0	2	6	10
6	-	-	-	-	-	-	-	-	-	-
7	15	0	2	2	6	2	2	2	6	5
8	15	2	4	1	4	0	2	2	6	5
9	-	-	-	-	-	-	-	-	-	-
10	15	2	2	1	6	2	0	2	8	5
11	-	-	-	-	-	-	-	-	-	-
12	-	-	-	-	-	-	-	-	-	-

13	15	2	2	2	6	0	2	2	6	5
14	15	2	2	2	6	2	0	2	6	5
15	-	-	-	-	-	-	-	-	-	-
16	15	0	2	2	4	2	2	2	6	10
17	15	0	2	1	6	2	2	2	6	10
18	-	-	-	-	-	-	-	-	-	-
19	15	2	2	1	4	2	0	2	8	5
20	-	-	-	-	-	-	-	-	-	-
21	15	0	4	2	6	0	2	2	6	5
22	15	0	4	1	4	2	0	2	6	5
23	15	0	2	2	6	0	2	2	6	5
24	15	2	2	1	4	2	2	2	8	10
25	15	2	2	1	6	2	0	2	6	10
26	15	2	2	1	4	0	2	2	8	10
27	15	0	2	2	6	2	0	2	6	10
